# Do positive relations with patients play a protective role for healthcare employees? Effects of patients' gratitude and support on nurses' burnout

**DOI:** 10.3389/fpsyg.2015.00470

**Published:** 2015-04-21

**Authors:** Daniela Converso, Barbara Loera, Sara Viotti, Mara Martini

**Affiliations:** ^1^Work and Organizational Psychology, Department of Psychology, University of TurinTurin, Italy; ^2^Psychometrics, Department of Psychology, University of TurinTurin, Italy

**Keywords:** patients' gratitude, patients' support, burnout, SEM, nurses

## Abstract

**Background:** A growing number of studies reveal that there are significant associations between a patient's perception of quality of care and a health professional's perceived quality of work life. Previous studies focused on the patients or on the workers. Alternatively, they center the discussion on either the negative or the positive effects, both on patients and care workers. This research work focuses on the positive relationship with patients—a possible resource for care workers.

**Method:** *Study 1*: A CFA was conducted to test the factorial structure and the tenure of the Italian version for patients of the Customer-initiated Support scale. *Study 2:* Using a multi-group path analysis, the effects of work characteristics and of the relationship with patients on burnout were tested in two different contexts: emergency and oncology ward.

**Results:** *Study 1*: The one-factor instrument shows good reliability, convergent, and divergent validity. *Study 2:* for oncology nurses cognitive demands, job autonomy, and support from patients have direct effects on emotional exhaustion and job autonomy; interactions between cognitive demands and patients' support have an effect on depersonalization. For emergency nurses cognitive demands and interactions between job autonomy and support from patients have effects on emotional exhaustion; job autonomy, patients support and gratitude have direct effects on personal accomplishment.

**Conclusions:** Results confirm expectations about the role of patients' support and gratitude in reducing nurses' burnout, with differences in the two contexts: emergency nurses show higher burnout and lower perception of positive relationship with patients, but present more intense protective effects of the interaction between job autonomy and support/gratitude. Suggestions can be offered to managers in developing interventions to promote “healthy organization” culture that consider jointly employees and patients' needs.

## Introduction

Despite the common practice to examine patients' and workers' well-being as separate entities, the majority of the findings in both fields implicitly confirm the interdependence, or even the reciprocity, between employees' health and patients' quality of care.

With regard to patients, the majority of previous research highlights how their positive relationship with physicians has “therapeutic potential” (Peabody, 1927) for them. Furthermore, research has shown that such a relationship can strengthen the adherence process of medical care (Zolnierek and Dimatteo, 2009; Velasco et al., 2014), enhance quality of life and patient satisfaction (Step et al., 2009), and contribute to a reduced time of hospitalization (Halbesleben and Rathert, 2008a). However, turning to the negatives, studies have shown that this relationship can also breed fear, uncertainty, and weak adherence (Johnston Roberts, 2002).

With regard to workers, there are distinguished studies relating to customer/patient stressors, i.e., aggressive behaviors or disproportionate requests. Such studies highlight how a negative relationship with patients can have harmful consequences for nurses and physicians, in terms of stress, burnout, and health complaints (Dormann and Zapf, 2004; Guglielmetti et al., 2014). Such negative consequences can lead to depersonalization and a detached, or even negative, behavior by workers. In general, it can additionally activate a spiral loss effect (Groth and Grandey, 2012), where the burnout of healthcare providers reduces the quality of care and patient safety (Halbesleben and Rathert, 2008b).

A smaller number of recent studies investigate the positive role of the relationship with customers/clients/patients and its effects for health providers. As scholars observe, it can in fact represent a job related resource for service workers, acting as a protective factor (Converso et al., 2009; Donoso et al., 2015). This helps to improve an “affective crossover” (Zimmermann et al., 2011) and lessen the effects of work overload or other work stressors.

Finally, a growing pool of studies examine patients' and workers' perceived health at the same time, revealing significant associations between patient dissatisfaction and their care providers' stress and burnout. They also indicate significant associations between perceived quality of care by patients and perceived quality of work life by employees in the same hospital ward (Vahey et al., 2004; Argentero et al., 2008; Ferrara et al., 2013; Weigl et al., 2013; Papastavrou et al., 2014). In other words, these studies reveal the systemic interaction between healthcare workers' psychological and physical health and patient outcomes. They suggest that no choice exists between providers' health and their recipients' quality of care. This is the perspective adopted by researchers of the Occupational Health Psychology (OHP). Moreover, it is exemplified by the definition of “Healthy Organizations” (Salanova et al., 2012). Those organizations that make systematic, planned, and proactive efforts to improve the employees and organizational health, promote a healthy working environment (i.e., a positive work-unit climate), healthy workers (i.e., higher levels of job satisfaction and reduced levels of job burnout of nurses and physicians) and organizational outcomes which express health (i.e., reduced time of hospitalization and quality of care perceived by patients).

Thus, the study of the reciprocity of quality of work life and quality of care, as well as the consequences of this on workers' and patients' health, have to be improved. We need to adopt the research perspective that jointly takes into account patients and healthcare providers of the same organizational unit and, as in this study, investigate the consequences of a positive relationship with patients for healthcare providers.

According to these premises, our study has been conducted on a sample of Italian nurses. This is so we can analyze the relationship between recipients and healthcare workers from the nurses' point of view. More specifically, our study aims to investigate whether the support perceived by patients (Zimmermann et al., 2011) and/or the gratitude expressed by them (Martini and Converso, 2012, 2014) has a direct effect on burnout. Furthermore, it aims to explore its indirect effect on reducing the negative effects of job demands or enhancing the positive effect of job resources.

In order to analyze whether the positive relationship with patients plays a role in the quality of life for health work professionals, we took into account some “traditional” job characteristics (psychological demands and job autonomy) that can have an impact on the perception of health at work.

Psychological demands refers to the degree to which tasks require workers to expend sustained mental effort in carrying out their duties. This kind of demand is particularly salient in the context of nursing. This is due to the high level of complexity of the job. Indeed, nurses are required to manage a high quantity of information, to use advanced technology and make decisions in an unpredictable environment. Recently, with regard to the nursing sector, factors such as the shortage of health sector resources or the introduction of new technologies are responsible for further intensified psychological demands. This is particularly demonstrated in terms of workload and an increased hectic pace (Greenglass, 2000; OECD, 2013). Moreover, extensive written studies highlight a strong, direct and positive relationship between job demands and burnout (van Vegchel et al., 2004; Borritz et al., 2005; Bakker and Demerouti, 2007; Crawford et al., 2010; Nahrgang et al., 2011).

Job autonomy is considered to be a central characteristic in the context of nursing (Finn, 2001). It refers to the extent to which a person is autonomous in task-related decisions, such as timing and method control (Karasek, 1985). Nurses are required to make important decisions during the care process. Thus, a lack of autonomy can leave them unable to accomplish their tasks properly. From an empirical point of view, evidence suggests that autonomy contributes to a decrease in emotional exhaustion and depersonalization. It also contributes to an increase in personal accomplishment (Lee and Ashforth, 1996; Rafferty et al., 2001).

With this, the positive side of the relationship with patients is considered. It appears from the patients' support and gratitude, expressed toward health professionals. Gratitude is considered to be a positive psychological characteristic. It is linked to a feeling of well-being (Toussaint and Friedman, 2008) and can create a positive spiral effect (McCullough et al., 2001), motivate pro-social behavior (Grant and Gino, 2010), and contribute toward cultivating social resources. Recently, gratitude has been considered “from the other side” of the relationship, taking into account the supportive effects of gratitude on the people who receive it (Martini and Converso, 2012, 2014).

In recent years, support by customer/patients has emerged as a novelty in the wide range of studies dedicated to social support at work (Johnson and Hall, 1988; Beehr et al., 2010). Here, it has been revealed as a powerful resource when “dealing with people” and a motivation for workers. It has been argued in fact that the positive relationship with recipients may develop the affective crossover between customers and employees, support providers, buffer stress and enhance well-being (Zimmermann et al., 2011).

According to the Job Demand Resources model (JD-R, Demerouti et al., 2001), job demands and job resources are the factors that affect workers psychological health. Job demands are physical, psychological, social, and organizational aspects of the job that require the workers to sustain efforts and have physiological and psychological costs, while job resources are aspects that help achieve work goals and reduce job demands. In this model, social support is an important resource to contain stress and burn-out (Karasek and Theorell, 1990; Demerouti et al., 2001). It can also have indirect effects: support can buffer the impact of job demands on job strain, including burnout (buffering hypothesis; van Emmerik et al., 2007) or enhance the positive action of resources on well-being at work, particularly in conditions of high demands (boostering hypothesis; Bakker et al., 2007).

In sum this study seeks to test the following hypotheses:

Hypothesis 1

There a significant, direct relationship between perceived support/gratitude expressed by patients and each of the three dimensions of burnout. More precisely:

- support and gratitude expressed by patients reduce emotional exhaustion and depersonalization
- support and gratitude expressed by patients increase personal accomplishment.

Hypothesis 2

Perceived support/gratitude expressed by patients buffers the effects of psychological demands in increasing burnout. Specifically:

- support and gratitude expressed by patients reduce the effects of psychological demands in increasing emotional exhaustion and depersonalization
- support and gratitude expressed by patients reduce the effects of psychological demands in decreasing personal accomplishment.

Hypothesis 3

Perceived support/gratitude expressed by patients enhances the effect of autonomy in reducing job burnout. Specifically:

- support and gratitude expressed by patients enhance the effect of autonomy in reducing emotional exhaustion and depersonalization
- support and gratitude expressed by patients enhance the effect of autonomy in increasing personal accomplishment.

Finally, as the organizational characteristics vary in relation to levels of emergency, intensity and technology (Tummers et al., 2002; van den Berg et al., 2006; Viotti et al., 2012; Loera et al., 2013), and in relation to patients' pathologies (Converso et al., 2009), the present research design included two hospital wards that are often considered for their specific risk factors for stress and burnout: the oncology unit (Escot et al., 2001) and the emergency unit (Hooper et al., 2010). These two units represent the different care processes in which health professionals are involved and can be considered as emblematic also for the different risk level of negative relationships (violence, aggressive behavior) with patients (Speroni et al., 2014).

## Materials and methods

### Ethical statement

The studies presented in this article conformed to the provisions of the Declaration of Helsinki in 1995, revised in Edinburgh 2000 (World Medical Association, 2001). All ethical guidelines were followed, as required for conducting human research, including adherence to the legal requirements of Italy. The research projects were approved by the Hospital Board of Directors of the hospitals involved in the study: Azienda Ospedaliera Universitaria San Giovanni Battista and Azienda Ospedaliera Ordine Mauriziano. Since there was no medical treatment or procedures causing participants psychological or social discomfort, additional ethical approval was not required. With the approval of the Hospital Board of Directors, department chiefs from each ward were asked for authorization to administer the questionnaire. The cover sheet clearly explained the research aim, the voluntary nature of participation, the anonymity of the data and the elaboration of the findings. Thus, returning the questionnaires implied consent. Participants volunteered in the research without receiving any reward.

### Procedure and participants

The research was divided into two studies. The first study was aimed at testing the factorial structure and the tenure of the Customers-initiated Support scale (Zimmermann et al., 2011). Exploring the impact of patients' support on workers' well-being had not yet been tested in the health sector. This enhanced the necessity of a dedicated instrument. Moreover, Zimmermann's scale was not validated in Italy but its adaptation is an innovative and useful tool for research that aims to test the effects of the positive side of the relations with patients on physicians and nurses well-being.

The second study focused on the role of perceived gratitude and support from patients on healthcare professionals' well-being, inversely measured as burnout. Study 2 tested the three hypotheses previously stated, in particular, the hypothesized moderation effect of the expressions of gratitude and support from patients on the relationship between job autonomy and psychological demands and burnout syndrome dimensions (Emotional Exhaustion; Depersonalization; Personal Accomplishment).

#### Study 1

The Customers-initiated support scale (Zimmermann et al., 2011) was originally developed to study positive customer behavior as a psychological resource for service employees. For this study, before developing our questionnaire, the scale was translated into Italian by the double-blind method and adapted to the sanitarian context, replacing “customer” with “patient” in each item.

For a comprehension pre-test, the Italian adapted version was proposed to 35 nurses. This was then inserted into the data collection tool. Moreover, in order to test the convergent and divergent validity of the Zimmermann's scale, the tool included a scale of perceived gratitude from patients (PGrate) and a scale of disproportionate customer expectations. The PGrate scale (Martini and Converso, 2014; Martini et al., in press) is a two dimensional scale (eight items) that measures the perception of patients' gratitude expression and of support provided by gratitude expressions. The Customer-related social stressors scale (Dormann and Zapf, 2004; Italian adaptation, Bakker et al., 2007; Taddei and Vanni, 2008; Guglielmetti et al., 2014) is a 28 item scale that measures four dimensions. Respectively, these are disproportionate customer expectations, customer verbal aggression, disliked customers, ambiguous customer expectations. In the data collection tool, only the relevant sub-dimensions of the two scales were considered and inserted: three items of gratitude expression (e.g., “Several patients express gratitude for the care we offer them”) and eight items of disproportionate customer expectations (e.g., item is “Our customers do not recognize the fact that we are very busy”).

The instrument was administered to a sample of 118 nurses in several wards of a Hospital of North-west Italy between April and December 2012. Nine subjects did not fully complete the questionnaire. Sample 1 was, therefore, made up of 109 healthcare workers. These were 78% women with an average age of 44 years (*SD* = 6.5). Of Sample 1, 55.1% were married, 25.5% were separated or divorced, and 19.4% were single. Of Sample 1, 96% were permanent employees and 4% were temporary employees.

Statistical analyses were carried out using the statistical packages SPSS 21 and Amos 20. In order to assess the psychometric proprieties of the scale of support from patients by Zimmermann et al. (2011), the descriptive analysis (*M, SD*; Asymmetry, Kurtosis) of each item of the scale was carried out. The normality of the items was checked by both the Kolmogorov–Smirnov and the Shapiro–Wilk tests, which are considered to be more powerful, according to Razali and Wah (2011). The reliability of the scale was assessed using the Cronbach's alpha, the corrected item-total correlations and the squared-multiple correlations. Their convergent and divergent validity was assessed through correlation analysis. A confirmatory factor analysis (CFA) was performed to verify the dimensionality, described by Zimmermann et al. (2011), of the Customers-initiated support scale. The Maximum Likelihood (ML) estimation method was employed. The fit of the model was assessed with the ratio of χ^2^ to the degrees of freedom (*df*), the Comparative Fit Index (CFI), the Goodness of Fit Index (GFI), and the Standardized Root Mean Square Residual (SRMR). According to Kline (1998), a χ^2^/*df* ratio of three or less indicates a good model fit. The GFI is a measure of the relative amount of variance and covariance explained by the model. The CFI indicates the amount of variation and covariation accounted by the model tested by comparing its fit with a baseline model's fit. For these two indices, values higher than 0.90 are considered to be indicators of a good model fit (Bentler, 1995; Hoyle, 1995). The SRMR is a measure of the mean absolute correlation residual that is the overall difference between the observed and predicted correlations. A value of the SRMR less than 0.05 indicates a good fit (Byrne, 1998; Diamantopoulos and Siguaw, 2000), less than 0.08 is considered acceptable (Hu and Bentler, 1999).

##### Descriptive single items analyses

As the descriptive analyses highlight, the five items of the Italian version for patients of the Customers-initiated support do not have normal distributions (Table 1). All item distributions show positive skewness (even if item 4 is not very sharpened) and negative kurtosis (except for item 5). Both the Kolmogorov–Smirnov and the Shapiro–Wilk's normality tests suggest a rejection of the hypothesis of normality of the distributions for all items.

**Table 1:** **Descriptive analysis of single items of the Italian version for patients of the Customers-initiated support**.

**S. No**.	**PGrate scale**	***M***	***DS***	**Skew**	**Kurt**	***KS***	***p***	***SW***	***p***
1	The patient adapted my working process *(I pazienti trovano adeguato il mio modo di lavorare)*	4.47	0.77	0.33	−0.44	0.30	0.000	0.74	0.000
2	The patient facilitated the service conversation through his/her previous knowledge *(I pazienti facilitano la comunicazione relativa al servizio di cura con le loro conoscenze precedenti)*	4.05	0.90	0.24	−0.03	0.31	0.000	0.82	0.000
3	The patient trusted in my competencies *(I pazienti si fidano delle mie competenze)*	4.65	0.80	0.10	−0.36	0.31	0.000	0.80	0.000
4	The patient explicitly valued my work effort *(I pazienti riconoscono esplicitamente l'impegno che metto nel lavoro)*	4.71	0.94	0.01	−0.51	0.26	0.000	0.85	0.000
5	The patient and I were on the same wave length *(I pazienti ed io siamo sulla stessa lunghezza d'onda)*	4.19	0.83	0.48	0.26	0.35	0.000	0.78	0.000

##### Confirmatory factor analysis

**Figure 1:**
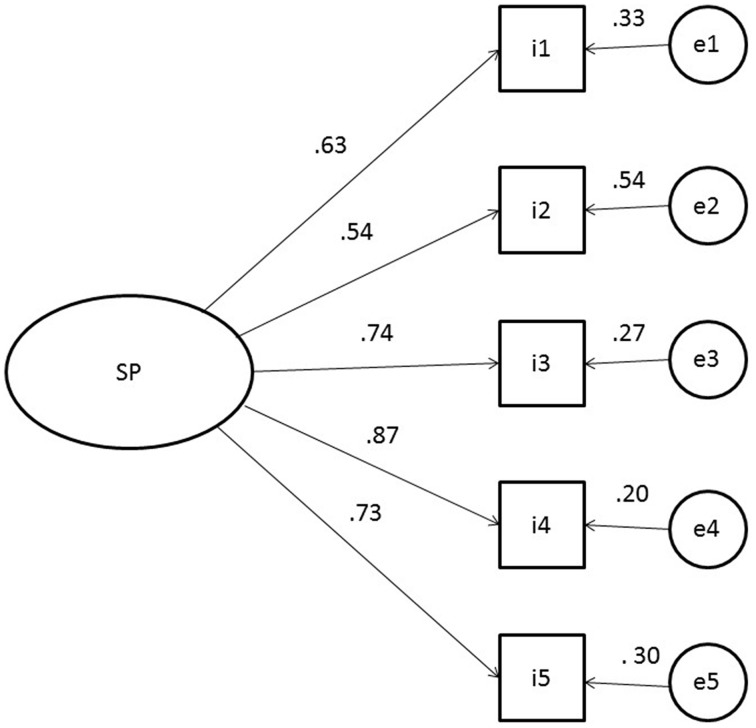
**Standardized parameter estimates for the factor structure of the Italian version for patients of the *Customer initiated support* scale**. Squares indicate the five items on the scale, circle represent the latent factor.

The results of the CFA showed then that the fit of the model tested was satisfactory: χ^2^ = 5.76, *df* = 5, χ ^2^/*df* = 1.15, CFI = 0.99, GFI = 0.98; SRMR = 0.03. All the item-factor relationships were significant (*p* = 0.000). The lowest loading value was obtained by item 2 (λ = 0.54) and the highest by item 4 (λ = 0.87). Figure 1 graphically shows the factorial structure of the scale.

##### Reliability

The scale showed an excellent internal consistency, since Cronbach's alpha reached the value of 0.84, coherently with Zimmermann et al. (2011) original study that showed value of 0.82. In addition (Table 2), item-scale correlation values were comprised between 0.60 (item 1) and 0.77 (item 4). This was definitely above the cut-off value of 0.40, indicated by Nunnally (1967).

**Table 2:** **Reliability analysis of the Italian version for patients of the Customers-initiated support**.

**S. No**.	**PGrate scale**	**Corrected item-total correlations**	**Squared-multiple correlations**	**Cronbach's alpha if item deleted**
1.	The patient adapted my working process *(I pazienti trovano adeguato il mio modo di lavorare)*	0.599	0.369	0.824
2.	The patient facilitated the service conversation through his/her previous knowledge *(I pazienti facilitano la comunicazione relativa al servizio di cura con le loro conoscenze precedenti)*	0.585	0.371	0.828
3.	The patient trusted in my competencies *(I pazienti si fidano delle mie competenze)*	0.642	0.463	0.812
4.	The patient explicitly valued my work effort *(I pazienti riconoscono esplicitamente l'impegno che metto nel lavoro)*	0.755	0.586	0.779
5.	The patient and I were on the same wavelength *(I pazienti ed io siamo sulla stessa lunghezza d'onda)*	0.668	0.467	0.805

##### Validity

The bivariate correlations results suggest evidence of both convergent and divergent validity for the Italian version for patients of the Customers-initiated support, as Table 3 highlights. The scale has a relevant positive correlation with the PGrate scale that measures gratitude expressions, a positive facet of patients' behavior. This is similar to Zimmermann's instrument and so, supports the hypothesis of convergent validity. It does not have any significant correlations with the disproportionate customer expectations (Dormann and Zapf, 2004) that express a critical dimension of the relationship with patients. This is consistent with the hypothesis of a low or zero correlation with divergent constructs.

**Table 3:** **Correlation of the Italian version for patients of the Customers-initiated support with other constructs, Means, Standard deviations, Alpha values**.

	**1**	**2**	**3**
1. Patients-initiated support	–		
2. Gratitude expressions	0.482^**^	–	
3. Disproportionate customer expectations	−0.141	−0.277^*^	–
M	4.48	4.39	2.58
SD	0.81	0.61	0.61
Alpha	0.84	0.85	0.89

* p < 0.001;

** *p < 0.05*.

#### Study 2

In Study 2, data were collected through a self-administered questionnaire. The instrument was administered to 210 nurses at two hospitals of North-west Italy between October 2013 and July 2014. Six questionnaires contained missing responses and so were omitted from the sample. The sample was, therefore, made up of 204 subjects. They were 77% women with an average age of 38 years (*SD* = 6.5). Of these subjects, 59.6% were married, 8.9% were separated or divorced, 31.5% were single. Furthermore, 88.6% of the subjects were permanent employees and 11.4% were temporary employees. On average, their occupation seniority in their sanitarian service was 9.92 (*SD* = 6.90) and they worked 5.18 (*SD* = 2.78) h a day. Of the participants, 95 (46.6%) were employed in the emergency unit and 109 (53.4%) in the oncology ward. In terms of socio-occupational characteristics, there were no significant differences between the two sub-samples.

##### Measures

In addition to the Italian version for patients of the Customers-initiated support scale (Zimmermann et al., 2011) and to the PGRate scale (Martini and Converso, 2014; Martini et al., in press), the questionnaire comprised scales aimed at measuring psychological demands, job autonomy, and job burnout. Psychological demands (three items) and job autonomy (three items) were two sub-scales of the Job Content Questionnaire (Karasek, 1985; Baldasseroni et al., 2001). Job burnout was measured by the 22 items of the Maslach Burnout Inventory for Human Service Sector (MBI-HSS, Maslach and Jackson, 1986; Italian version, Sirigatti and Stefanile, 1993; Italian revision, Loera et al., 2014). This was made up of the three sub-scales of emotional exhaustion (EE, nine items), depersonalization (DP, five items) and personal accomplishment (PA, eight items).

A structured equation model (SEM) was hypothesized in order to estimate the direct effects of job autonomy and psychological demands on burn-out (EE, DP, and PA), as well as the indirect effects of the interaction with a perceived patient's gratitude and support. To test the model, a multi-group path analysis with AMOS 20 was performed on the two sub-samples of nurses. The method of estimation used was Maximum Likelihood (ML).

**Figure 2:**
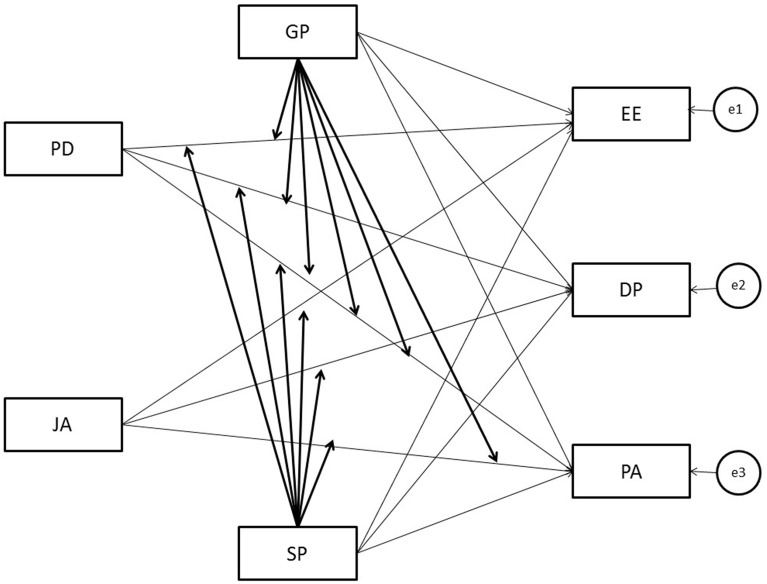
**Representation of the model tested**. CD, Psychological Demands; JA, Job decision-making Autonomy; GP, Gratitude from Patients; SP, Support from Patients; EE, Emotional Exhaustion; D, Depersonalization; PA, Personal Accomplishment.

As shown in Figure 2, the models tested included direct paths from eight exogenous factors to three endogenous factors (emotional exhaustion, depersonalization and personal accomplishment). Four exogenous factors represent; (a) the main terms of work characteristics (job decision-making autonomy, JA; psychological demands, PD), (b) the positive side of the relationship (gratitude from patients, GP; support from patients, SP). Two exogenous factors represent the cross-products between PD^*^GP and PD^*^SP. They were entered in order to test the buffering effects of, respectively, the gratitude from patients and the support from patients in the relationship between psychological demands and the three burnout sub-dimensions. Similarly, to test the booster effects of the gratitude from patients and the support from patients in the relationship between job autonomy and the three burnout sub-dimensions. Thus, two exogenous variables that represent the cross-products between JA^*^GP and JA^*^SP were included. The main terms of the work characteristics and of the positive relationship could correlate (i.e., JA-PD, JA-GP, JA-SP, PD-GP, PD-SP, GA-SP). Furthermore, each interactional term could correlate with the two main terms that it consisted of (i.e., PD^*^GP-PD, PD^*^GP-GP, PD^*^GP-PD, PD^*^SP-SP, JA^*^GP-GP, JA^*^GP-JA, JA^*^SP-SP, JA^*^SP-JA). Finally, three path coefficients were also added between the outcomes (i.e., EE→DP, EE→PA, DP→PA).

The overall fit of the model was assessed with the ratio of χ^2^ to the degrees of freedom (*df*), the Comparative Fit Index (CFI), the Goodness of Fit Index (GFI), and the Standardized Root Mean Square Residual (SRMR).

##### Data analyses

The scales used in Study 2 showed satisfying homogeneity between the items and good reliability, except for psychological demands and depersonalization. However, both of them are considered to be reliable and valid as they are commonly used in research studies. In particular, among the three dimensions of the MBI, DP usually shows the lower reliability, even if it can vary in different samples (Aguayo et al., 2011).

Scores of each construct were calculated by medium values of the relative items. Table 4 reports the description of the score for the two groups.

**Table 4:**
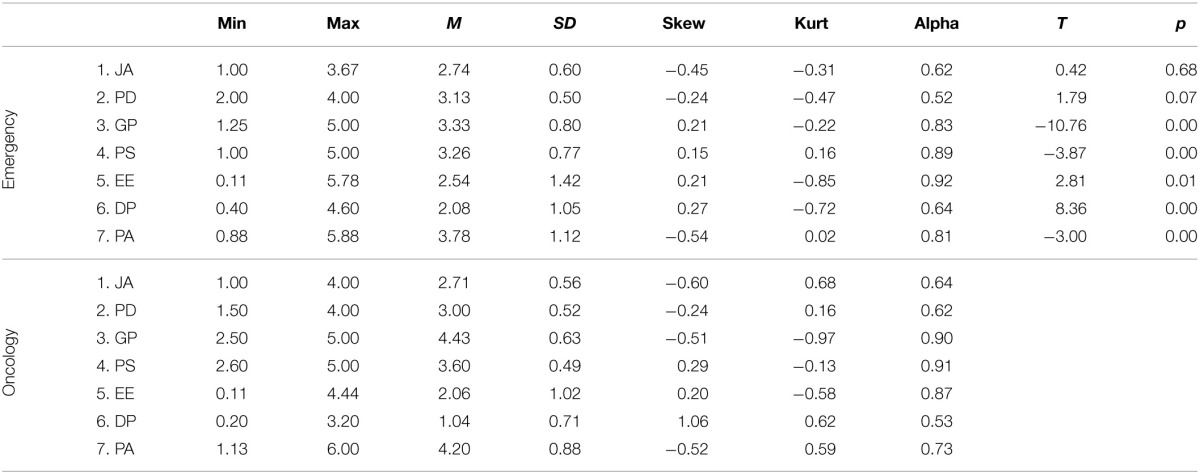
**Construct scores description, alpha values and differences between scores in Emergency (*N* = 95) and Oncology (*N* = 109)**.

*JA, Job Autonomy; PD, Psychological Demands; GP, Gratitude from Patients; SP, Support from Patients; EE, Emotional exhaustion; DP, Depersonalization; PA, Personal accomplishment. Standard errors for Skewness and Kurtosis are, respectively, 0.25 and 0.49. GDL for the T-tests are 202*.

##### T-test

The two groups showed different medium values for all analyzed scales, except for autonomy. More precisely, health professionals who worked in the emergency unit have higher psychological demands, EE and DP than professionals of the oncology ward. The latter group declared a higher level of gratitude expression, patients-initiated support and PA, than the former.

##### Correlations

Additionally, the relationships between constructs were partially different for the two groups (Table 5). In the oncology ward, autonomy was correlated with Patients-initiated support and with DP, patients-initiated support was correlated with EE and DP, PA was correlated with EE and DP. This is contrary to the emergency room. Furthermore, in the emergency room, gratitude correlates with EE and with DP. This is contrary to the oncology ward.

**Table 5:** **Correlations between constructs**.

	**1**	**2**	**3**	**4**	**5**	**6**	**7**
1. JA		0.080	0.368^*^	0.144	−0.211^*^	−0.171	0.374^*^
2. PD	−0.164		0.057	−0.198	0.273^*^	0.182	−0.145
3. GP	0.195^*^	0.067		0.440^*^	−0.361^*^	−0.274^*^	0.389^*^
4. PS	0.230^*^	0.097	0.307^*^		−0.173	−0.053	0.373^*^
5. EE	−0.380^*^	0.468^*^	−0.043	−0.278^*^		0.629^*^	−0.120
6. DP	−0.313^*^	0.042	−0.156	−0.253^*^	0.273^*^		−0.128
7. PA	0.307^*^	0.056	0.304^*^	0.587^*^	−0.266^*^	−0.252	

*Emergency sub-sample above the matrix diagonal. JA, Job Autonomy; PD, Psychological Demands; GP, Gratitude from Patients; SP, Support from Patients; EE, Emotional exhaustion; DP, Depersonalization; PA, Personal accomplishment*.

* **p* < 0.001*.

##### Multi-group path analysis

The model tested (Figure 2) showed an acceptable fit: χ^2^ = 73.48, *df* = 28, χ^2^/*df* = 2.62, CFI = 0.87, GFI = 0.95; SRMR = 0.07. Tables 6, 7 reported, for the considered two sub-samples, the estimates of the path coefficients from the main and interactional effects of work characteristics to each burnout dimension.

**Table 6:** **Maximum likelihood estimates of path coefficients for path analysis among oncology nurses**.

**Predictors**	**Emotional exhaustion**			**Depersonalization**			**Personal accomplishment**		
	**SPC**	***SE***	***p***	**SPC**	**SE**	***p***	**SPC**	***SE***	***p***
PD	0.456	0.085	0.000^*^	−0.037	0.078	0.732	0.110	0.084	0.245
JA	−0.242	0.079	0.002^*^	−0.228	0.067	0.014^*^	0.127	0.074	0.128
GP	−0.068	0.080	0.387	−0.103	0.065	0.257	0.108	0.070	0.180
SP	−0.289	0.082	0.000^*^	−0.149	0.071	0.123	0.472	0.077	0.000^*^
PD*GP	0.010	0.090	0.902	−0.112	0.073	0.235	−0.063	0.079	0.451
PD*SP	0.035	0.097	0.637	0.231	0.078	0.007^*^	0.031	0.087	0.694
JA*GP	0.133	0.078	0.071	0.014	0.064	0.873	0.038	0.069	0.616
JA*SP	−0.092	0.081	0.210	0.018	0.066	0.835	−0.061	0.071	0.421

*PD, Psychological Demands; JA, Job Autonomy; GP, Gratitude from Patients; SP, Support from Patients; SPC, Standardized Path Coefficient; SE, Standard Error*.

* *p < 0.05*.

**Table 7:** **Maximum likelihood estimates of path coefficients for path analysis among emergency nurses**.

**Predictors**	**Emotional exhaustion**			**Depersonalization**			**Personal accomplishment**		
	**SPC**	***SE***	***p***	**SPC**	**SE**	***p***	**SPC**	***SE***	***p***
PD	0.288^*^	0.126^*^	0.000^*^	0.062	0.091	0.470	−0.174	0.104	0.059
JA	−0.033	0.140	0.737	−0.040	0.096	0.667	0.257	0.109	0.009^*^
GP	−0.335^*^	0.147^*^	0.000^*^	−0.063	0.107	0.528	0.264	0.122	0.014^*^
SP	−0.024	0.146	0.811	0.089	0.099	0.346	0.227	0.114	0.024^*^
PD^*^GP	0.022	0.125	0.791	−0.032	0.085	0.682	−0.201	0.097	0.016^*^
PD^*^SP	0.131	0.106	0.118	−0.121	0.073	0.132	0.059	0.085	0.496
JA^*^GP	0.140	0.127	0.115	−0.048	0.087	0.567	−0.055	0.100	0.542
JA^*^SP	−0.296^*^	0.102^*^	0.000^*^	−0.025	0.074	0.779	−0.045	0.084	0.630

*PD, Psychological Demands; JA, Job Autonomy; GP, G ratitude from Patients; SP, Support from Patients; SPC, Standardized Path Coefficient; SE, Standard Error*.

* *p < 0.05*.

Among the oncology nurses (Table 6), the standardized path coefficient (SPC) indicated that EE has a significant relationship with three of the four dimensions considered. It positively related to the main term of psychological demands (SPC = 0.456, *p* = 0.000). However, it negatively related to the main terms of job autonomy (SPC = −0.242, *p* = 0.002) and support from the patients (SPC = −0.289, *p* = 0.000). DP was found to be negatively associated only to the main terms of job autonomy (SPC = −0.228). No significant path coefficients were found considering PA. With regard to interactions, only the path coefficient from the cross-product between psychological demands and support from patients to DP was associated to a significant *p*-value (SPC = 0.231, *p* = 0.007).

Concerning the emergency nurses (Table 7), EE was significantly predicted by psychological demands (SPC = −0.288, *p* = 0.002). No significant path coefficients were found between the main terms of the work characteristics and DP. PA was found positively associated to the main terms of job autonomy (SPC = 0.257, *p* = 0.002), support (SPC = 0.288, *p* = 0.002) and gratitude (SPC = 0.227, *p* = 0.024) from patients. Concerning the interactions, only the path coefficient from the cross-product between autonomy and support from patients to EE was found to be significant (SPC = −0.296, *p* = 0.000).

## Discussion

The positive role of the relationship with patients for nurses' well-being had rarely been explored in previous studies. This paper aimed at measuring some salient dimensions of the relationship caregiver-recipients and their impact on workers' well-being, within emergency and oncology nurses.

The first study contributed to the validation of the scale of support received from patients. The factorial analysis confirmed that the Zimmermann's items (Zimmermann et al., 2011) generate a one-dimensional measure of patients' support. This coherently correlates with the perceived gratitude expressed by patients (Martini and Converso, 2014) and has no correlation with the divergent constructs, such as disproportionate customer expectations (Dormann and Zapf, 2004).

The second study was oriented by three main hypotheses. The first one concerns the existence of a significant, direct relationship, between perceived support/gratitude expressed by patients and each of the three dimensions of burnout. The second the existence of a buffering effect of perceived support/gratitude expressed by patients on the relation between psychological demands and burnout. The third one the existence of a boostering effect (Bakker et al., 2007) of perceived support/gratitude expressed by patients on the relation between autonomy and job burnout.

Compared with oncology nurses, emergency nurses expressed a higher level of psychological burden, emotional exhaustion, depersonalization, and in general, a lower sensibility to the positive relationship with patients. Oncology nurses showed a higher perception of gratitude expression and patients-initiated support and declared a higher personal accomplishment than emergency nurses.

With regard to the first hypotheses, we found significant direct effects of patient-initiated support and gratitude on nurses' burnout, but were limited to EE and PA, with different patterns in the two units. With the oncology nurses, only the relation between patients' support, EE and PA emerged. In the emergency group, support had a relation with PA, while gratitude was related to both EE and PA.

Moreover, with regard to the second hypotheses, significant interactions were found for oncology nurses between psychological demands and patient-initiated support on DP. In presence of support, DP increased in the function of psychological demands but its level remained lower than in the non-supported group, in which DP was high and did not vary in relation to psychological demands.

Among emergency nurses, a significant interaction was found between psychological demands and gratitude on PA. As the psychological demands grew, the PA diminished. However, when gratitude was expressed, personal accomplishment was significantly higher than in the low gratitude expressed situation.

Finally, with regard to the third hypotheses, no significant interactions were found among oncology nurses. On the contrary, among the emergency nurses, a significant interaction was found between job autonomy and support from patients on EE. In conditions of low support, there was almost no gap in EE scores between nurses who had high and low levels of autonomy, respectively. Moreover, in conditions of high support, the levels of EE were significantly higher among nurses who perceived low autonomy, in comparison with those who perceived high autonomy. These results are consistent with the booster hypothesis. It can be argued that these results are specific for an emergency unit, where autonomy represents a crucial resource that can be enhanced by patient collaboration. For example, when decisions have to be made quickly. If patients provide information and collaborate, this could make it easier to choose the best form of treatment, any tests required, etc., and, thus, the situation would be less stressful.

In sum: the first hypotheses was confirmed for two of the three dimensions of burnout (EE, PA), with different patterns for the two units; the second hypotheses was confirmed, with different patterns for the two units; the third hypotheses was partially confirmed and only for the emergency nurses.

## Limitations and future studies

This study has some limitations: firstly, the reduced dimension of the samples—participants are recruited in two hospitals in Northern Italy—and secondly, the preeminence of the female gender (typical of the nursing profession). Moreover, this study is cross sectional and may suffer of some methods effects due to the fact that the respondents providing the measure of the predictor and criterion variable are the same persons (Campbell and Fiske, 1959; Podsakoff et al., 2003). Future work would enlarge the respondents, control the gendering effect, and consider a wider number and typology of hospital wards.

## Conclusions and practical implications

The results confirm the general expectations of the role of patients' support and perceived gratitude, although there are differences between the two contexts (emergency/oncology wards). There are differences in how the relationship is perceived and concerning the three dimensions of burnout, that are consistent with previous studies. In contexts characterized by greater contact with technology and technical expertise—where the work shifts and workload are presumably more demanding and the relationship with patients is definitely shorter and more superficial—nurses show higher levels of burnout and have a less positive perception of the relationship with patients. Nonetheless, the interactions between psychological demands, job autonomy and support/gratitude were significant. They demonstrated how a positive relationship with patients is beneficial, especially for nurses on emergency wards.

In organizational practice, reflecting on the protective value of the positive side of the relationship with patients (Donoso et al., 2015) would guide managers in developing organizational interventions to foster workers' capability to manage and prevent negative patient behaviors, to evaluate and consider customers as resources. Furthermore, reflection would encourage interventions aimed at balancing customers-related social stressors by supportive customer interactions or through other social support resources (i.e., co-workers or supervisors) to compensate patients' behavior (Dudenhöffer and Dormann, 2013). Research focused on the systemic relationship between workers and patients would contribute to the promotion of a “healthy organization” culture, where employees' and patients' needs are not considered separately.

### Conflict of interest statement

The authors declare that the research was conducted in the absence of any commercial or financial relationships that could be construed as a potential conflict of interest.
